# Surface Properties and Permeability to Calcium Chloride of *Fagus sylvatica* and *Quercus petraea* Leaves of Different Canopy Heights

**DOI:** 10.3389/fpls.2018.00494

**Published:** 2018-04-18

**Authors:** Héctor A. Bahamonde, Luis Gil, Victoria Fernández

**Affiliations:** ^1^Instituto Nacional de Tecnología Agropecuaria, Buenos Aires, Argentina; ^2^Department of Natural Resources, Universidad Nacional de la Patagonia Austral, Río Gallegos, Argentina; ^3^Forest Genetics and Ecophysiology Research Group, School of Forest Engineering, Technical University of Madrid, Madrid, Spain

**Keywords:** cuticle, *Fagus sylvatica*, *Quercus petraea*, foliar absorption, plant surfaces, waxes, wettability

## Abstract

Plant surfaces have a considerable degree of chemical and physical variability also in relation to different environmental conditions, organs and state of development. The potential changes on plant surface properties in association with environmental variations have been little explored so far. Using two model tree species (i.e., *Quercus petraea*, sessile oak and *Fagus sylvatica*, beech) growing in ‘Montejo de la Sierra Forest,’ we examined various traits of the abaxial and adaxial surface of leaves of both species collected at a height of approximately 15 m (top canopy), versus 3.5–5.5 m for beech and sessile oak, lower canopy leaves. Leaf surface ultra-structure was analyzed by scanning and transmission electron microscopy, and the surface free energy and related parameter were estimated after measuring drops of 3 liquids with different degrees of polarity and apolarity. The permeability of the adaxial and abaxial surface of top and bottom canopy leaves to CaCl_2_ was estimated by depositing 2 drops of 3–4 μl per cm^2^ and comparing the concentration of Ca in leaf tissues 24 h after treatment, and also Ca and Cl concentrations in the washing liquid. Higher Ca concentrations were recorded after the application of CaCl_2_ drops onto the veins and adaxial blade of top canopy beech leaves, while no significant evidence for foliar Ca absorption was gained with sessile oak leaves. Surprisingly, high amounts of Cl were recovered after washing untreated, top canopy beach and sessile oak leaves with deionised water, a phenomenon which was not traced to occur on lower canopy leaves of both species. It is concluded that the surface of the two species analyzed is heterogeneous in nature and may have areas favoring the absorption of water and solutes as observed for the veins of beech leaves.

## Introduction

Plant surfaces are important for protecting organs against multiple biotic and abiotic stress factors (Serrano et al., 2014; Domínguez et al., 2017), being of paramount importance for preventing the uncontrolled loss of water (Riederer and Schreiber, 2001).

The surface of the epidermal cells (also including trichomes or guard cells) of most aerial plant organs is covered with an extracellular layer named cuticle, that is laid down at early developmental stages (Ingram and Nawrath, 2017; Petit et al., 2017). The cuticular membrane is traditionally defined as the non-living covering produced by the epidermis of some non-vascular land plants and the primary aerial organs of all vascular plants (Niklas et al., 2017). More recently, it has become apparent that it is a specialized (but nevertheless integral) part of the primary cell wall (Guzmán et al., 2014; Fernández et al., 2016), analogous in many regards to a lignified or a suberized secondary cell wall (Niklas et al., 2017). This layer plays a crucial eco-physiological role when stomata are closed, and it is assumed that leaves of drought tolerant species may have a lower cuticular permeability than species from more mesic habitats (Schuster et al., 2017).

The absorption of liquids via the foliage has long being recognized (e.g., Franke, 1967; Fernández and Eichert, 2009) and is a phenomenon of eco-physiological and agronomic significance (Fernández and Brown, 2013; Fernández et al., 2017). Several studies highlighted the importance of foliar absorption of liquid water (e.g., Oliveira et al., 2005; Breshears et al., 2008; Cassana and Dillenburg, 2013; Fernández et al., 2014b), fog (Limm and Dawson, 2010; Eller et al., 2013, 2016; Berry et al., 2014), dew (Munné-Bosch et al., 1999; Pina et al., 2016), foliar fertilizers (Fernández and Eichert, 2009; Eichert and Fernández, 2012) or biostimulants (Brown and Saa, 2015). The wettability and adhesion or repellence of water/solute drops onto plant surfaces is preliminary step that may either facilitate or impede foliar absorption (Fernández and Brown, 2013; Fernández et al., 2017).

*Fagus sylvatica* L. (European beech) and *Quercus petraea* (Matt.) Liebl. (Sessile oak) are the most common native, broad-leaf species of European temperate forests that coexist in mixed stands despite their contrasting shade tolerance (Mette et al., 2013; Petritan et al., 2014). The competition between these two species is high (Rohner et al., 2012) and is usually driven by the higher shade tolerance of beech compared to the higher light requirements of sessile oak (Collet et al., 1997; Stancioiu and O’Hara, 2006; Petritan et al., 2009).

In southern Europe, these two species co-exist in ‘Montejo de la Sierra Forest’ which represents one of the southern limits of the distribution of beech trees in this continent. Both species have different crown architecture, with sessile oak having a large foliage volume in the upper canopy that severely reduces irradiation at the lower canopy, and beech trees having a more open top canopy that enables radiation to reach middle canopy leaves that are preserved at lower tree heights (Cano et al., 2013). Thereby, leaves located at the base of the crown in sessile oak trees have been found to be physiologically similar to lower canopy beech leaves (Cano et al., 2013). Several studies related to water use and gas exchange of both species have been carried out in this forest. For instance, Aranda et al. (2000) concluded that sessile oak was more capable of coping with drought stress under extreme summer soil dryness than European beech. Furthermore, Aranda et al. (2005) reported that beech was more sensitive to a loss of the hydraulic conductance than sessile oak. When comparing sun-exposed versus shade leaves of both species of trees growing in ‘Montejo de la Sierra Forest,’ Cano et al. (2013) observed that moderate drought decreased the photosynthetic rate of top and low canopy leaves of beech much more than in leaves located in the mid canopy. By contrast, for sessile oak, moderate drought decreased the photosynthetic rate of lower canopy leaves more than in upper canopy leaves.

The process of foliar absorption of water and solutes will be first influenced by the interactions between drops and plant surfaces, prior to the transport of electrolytes through the epidermis (Fernández et al., 2017). A preliminary characterization of plant surface properties can be obtained by calculating the surface free energy and related parameters as described by Fernández and Khayet (2015). These values can provide information for example, on the potential interactions between plant surfaces and aqueous solutions, and may be helpful for discriminating between potentially surface-permeable or -impermeable organs and/or species in the future (Fernández et al., 2017).

Based on these preliminary investigations, the goal of this study was to examine the properties of the adaxial and abaxial surface of upper versus lower canopy leaves of beech and sessile oak trees growing at ‘Montejo de la Sierra Forest.’ Assuming the importance of the upper and lower leaf epidermis as main barrier for preventing water loss, and also for potentially facilitating the absorption of water and improving water economy during drought spells, the following hypotheses were tested: (i) the abaxial and adaxial side of leaves collected from the top versus bottom of the canopy varies in response to the prevailing environmental conditions during growth, (ii) the surface of leaves may be heterogeneous in nature and contain areas that may facilitate the absorption of water and solutes.

## Materials and Methods

### Plant Material and Experimental Conditions

Trials were performed with *Fagus sylvatica* L. (European beech) and *Quercus petraea* (Matt.) Liebl. (sessile oak) leaves of trees grown in a mature mixed stand located at the Biosphere Reserve of ‘Sierra del Rincón’ (also known as ‘Montejo de la Sierra Forest,’ 41°7′N, 3°30′W). The annual precipitations in the site averaged 950 mm between 1994 and 2010, but only a mean of 10% fallen during summer for the same period (Cano et al., 2013). With a depth of 1.1 m the soil is categorized as a Luvisol Orthodystric according to the FAO (2006) classification.

Leaves of three co-dominant trees of each species were collected by climbing a 25 m tower installed in the middle of the forest. Samples were taken from 2 different canopy layers, i.e.: ‘Top’ corresponding to maximum values of direct irradiation of leaves located at a height of 15.5–16 m for both species; and ‘Bottom’ canopy leaves which were collected at a height of 3.5 and 5.5 m for beech and sessile oak, respectively. At the moment of sampling (midday of July 18th, 2016), the total radiation at top and lower canopy positions (10 measurements per each place) was measured with a Li-185B Quantum/Radiometer/Photometer (LI-COR, Lincoln, NE, United States). The values determined for both species were 1260 and 125 μmol quanta m^-2^ s^-1^, at the top and bottom of the tree canopy, respectively. A dew point scanner (PCE-DPT 1, PCE Instruments, Spain) was used for measuring the temperature (T) and relative humidity (R.H.) values at the time of sample collection (approximately 12 p.m.). The mean values recorded were: 37.4 ± 0.8°C T and 19.9 ± 1.4% R.H. at the top of the canopy, 31.3 ± 0.6°C and 26.1 ± 0.8% R.H. at 5.5 m high (were the first layer of sessile oak leaves appeared), and 29.2 ± 0.2°C and 29.7 ± 1.0% R.H. at a height of 3.5 m, where lower canopy beech leaves were sampled. For the 2 different canopy layers, 10 shoots at least having 5 fully-expanded, healthy leaves were collected per tree. Shoots were subsequently kept under moist and cold conditions, and were rapidly transported to the laboratory for the development of trials.

Leaf thickness was measured with a micrometer (4000/F Baxlo, Spain, with 30 repetitions). The area, fresh and dry (2 days at 70°C) weight of 30 individual leaves was determined, expressing the specific leaf area (SLA) as the leaf surface to dry weight ratio (in m^2^ kg^-1^).

### Contact Angle Measurements and Surface Free Energy Calculations

Advancing contact angles of drops of double-distilled water, glycerol (Sigma-Aldrich) and diiodomethane (Sigma-Aldrich) were measured at 20°C, using a Drop Shape Analysis System (DSA 100, Krüss, Germany). Approximately, 2 μl drops of each liquid were deposited onto the adaxial and abaxial surface of beach and sessile oak leaves (onto green leaf blade areas and major leaf veins of the abaxial side which had a smooth concave topography), using a dosing system holding a 1 mL syringe (0.5 mm diameter needle). Side view images of the drops were captured at a rate of 10 frames s^-1^. Contact angles were automatically calculated by fitting the captured drop shape to the one calculated from the Young–Laplace equation.

For all the leaf surfaces analyzed, the total surface free energy (*γ*), its components [i.e., the Lifshitz-van der Waals (*γ*_s_^LW^) and acid-base (*γ*_s_^AB^; *γ*^+^ and *γ*_s_^-^)], and surface polarity were calculated considering the surface tension components of water (*γ*_l_ = 72.80 mJ m^-2^, *γ*_l_^LW^ = 21.80 mJ m^-2^, *γ*_l_^+^ = *γ*_l_^-^ = 25.50 mJ m^-2^), glycerol (*γ*_l_ = 63.70 mJ m^-2^, *γ*_l_^LW^ = 33.63 mJ m^-2^, *γ*_l_^+^ = 8.41 mJ m^-2^, *γ*_l_^-^ = 31.16 mJ m^-2^) and diiodomethane (*γ*_l_ = *γ*_l_^LW^ = 50.80 mJ m^-2^, *γ*_l_^+^ = 0.56 mJ m^-2^, *γ*_l_^-^ = 0 mJ m^-2^, Fernández and Khayet, 2015).

### Extraction of Soluble Cuticular Lipids

Fully expanded, intact leaves of beech and sessile oak from different canopy heights were selected and the mid vein was removed prior to sectioning leaves in large pieces which were subsequently weighed and scanned for surface area calculations. Leaf pieces were then individually held with pincers and either their adaxial or abaxial side was carefully washed with flushes of chloroform (Sigma-Aldrich) using a Pasteur pipette (6 pipette flushes of approximately 1.7 ml each per leaf piece). Chloroform extracts were then evaporated until dryness in watch glasses under a laboratory fume cupboard. The amount of soluble cuticular lipids (with 2 repetitions per treatment) was consequently expressed on a leaf surface area and dry weight (D.W.) basis.

### Electron Microscopy

Healthy, intact leaves were examined by scanning and transmission electron microscopy (SEM and TEM, respectively).

Gold-sputtered, adaxial beech and sessile oak leaf surfaces were directly observed with a Hitachi S-3400 N (Tokyo, Japan) SEM.

For TEM and optical microscopy, beech and sessile oak leaves were cut into 4 mm^2^ pieces and fixed in 2.5% glutaraldehyde-4% paraformaldehyde (both from Electron Microscopy Sciences (EMS), Hatfield, PA, United States) for 6 h at 4°C, rinsed in ice-cold phosphate buffer, pH 7.2, four times within a period of 6 h and left overnight. Tissues were post-fixed in a 1:1 2% aqueous osmium tetroxide (TAAB Laboratories, Berkshire, United Kingdom) and 3% aqueous potassium ferrocyanide (Sigma-Aldrich) solution for 1.5 h. They were then washed with distilled water (x3), dehydrated in a graded series of 30, 50, 70, 80, 90, 95, and 100% acetone (x2, 15 min each concentration) and embedded in acetone-Spurr’s resin (TAAB Laboratories) solutions (3:1, 2h; 1:1; 2h; 1:3; 3h) and in pure resin for the whole night at approximately 25°C. Embedding was finally carried out in blocks which were incubated at 70°C for 3 days for complete polymerization.

For TEM, ultra-thin sections were cut with an ultra-microtome. They were subsequently mounted on nickel grids and post-stained with Reynolds lead citrate (EMS) for 5 min prior to observation with a Jeol 1010 electron microscope (Tokyo, Japan) at 80 kV, equipped with a CCD megaview camera.

On the other hand, semi-thin leaf cross-sections were cut, mounted in microscope slides and stained with toloudine blue prior to observation with an epifluorecence microscope (Axioplan-2, Zeiss, Germany).

The thickness of the cuticle and of the epidermal cell wall of adaxial and abaxial beech and sessile oak leaves were measured on TEM micrographs using ImageJ1.45s (National Institutes of Health, Bethesda, MD, United States) software. Measurements were carried out at periclinal areas on 10 micrographs per sample, with 20 repetitions. Adaxial and abaxial surface features including stomatal densities were assessed by image analysis of SEM micrographs (ImageJ 1.45s).

### Foliar Absorption of Calcium Chloride

For assessing the permeability of beech and sessile oak leaf surfaces to solutes and water, a foliar application experiment was conducted under laboratory using 150 mM CaCl_2_ as a tracer. This chemical has a low point of deliquescence (32%, Schönherr, 2000) and since it is an electrolyte, it is assumed that it will penetrate the cuticle via the so-called “polar pathway” which has also been associated with the mechanisms of water diffusion (Schreiber and Schönherr, 2009).

**FIGURE 1:**
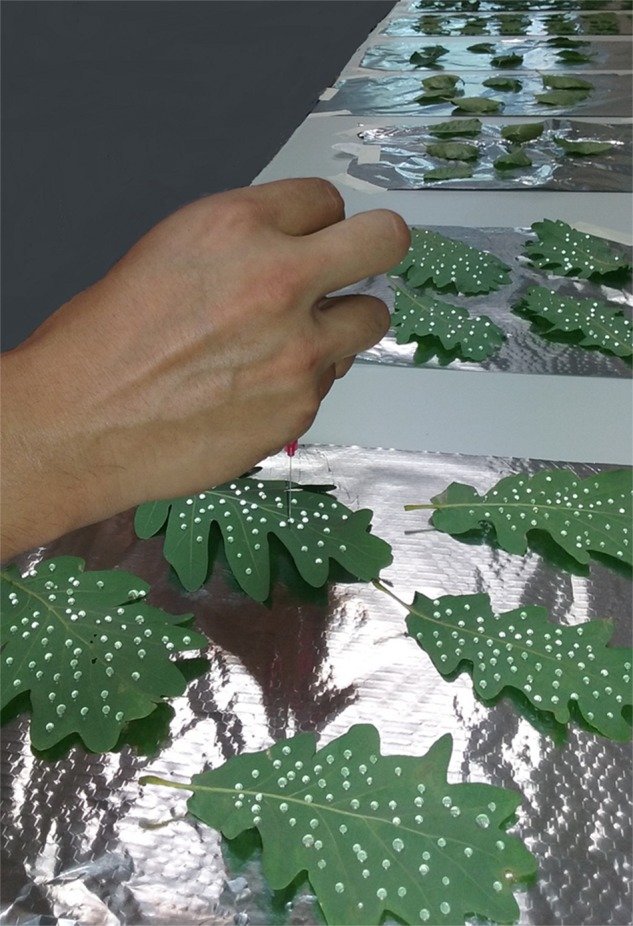
Deposition of 150 mM CaCl_2_ drops onto beech and sessile oak adaxial and abaxial plant surfaces using a micro-syringe to carry out a foliar absorption trial.

For carrying out a foliar absorption trial, undamaged leaves were selected, weighed and scanned for surface area determination. An amount of 2 drops of approximately 3–4 μl CaCl_2_ per square cm of leaf surface was subsequently applied, using a micro-syringe (**Figure 1**). The number of CaCl_2_ drops per leaf was calculated on a leaf area basis, considering the application of 2 drops per cm^2^, with 3 repetitions per treatment. Due to technical constrains for depositing the drops onto the veins and in light of the anatomic observations and surface free energy values previously determined, CaCl_2_ drops were only applied onto the adaxial surface of the major veins of beech leaves from the upper canopy since it was unfeasible to do this on the smaller-sized, lower canopy leaves. Leaves were placed onto aluminum foil with the surface to be treated being in direct contact with the surrounding air (**Figure 1**). Then drops were carefully deposited onto the surfaces with three repetitions per treatment (*N* = 10, for lower canopy beech leaves and *N* = 2 for all sessile oak treatments and top canopy beech leaves), including also a set of untreated beech and sessile oak leaves. Calcium chloride treated and untreated leaves were left standing for 24 h in the laboratory (at 19–21°C and 34–40% R.H.). After 24 h, treated and untreated leaf surfaces were carefully washed with flushes of deionised water using a Pasteur pipette to reach a total washing liquid volume of 50 ml. The Ca and Cl concentrations recovered after leaf surface washing with deionised water were determined by inductively coupled plasma (ICP-OES for Ca; Optima 3000, Perkin-Elmer) and ion chromatography (Cl; Metrhom Switzerland). Additionally, the Ca concentration of dried (70°C, for 2 days), ground Ca-treated and untreated leaf tissues was also measured by ICP-OES.

### Data Analysis

Statistical analyses were carried out using SPSS 15.0 (SPSS Inc., Chicago, IL, United States). Leaf structural parameters, contact angles, and wax, Ca, and Cl concentrations were analyzed by one-way ANOVA. Duncan’s Multiple Range Tests were carried out to test differences between factors when *F*-values were significant (*P* < 0.05).

## Results

### Leaf Characteristics as Affected by Canopy Position

The specific leaf area, leaf thickness, area and stomatal density of beech and sessile oak leaves collected from the top or bottom of the tree canopy are shown in **Table 1**. Beech leaves collected from the top of the crown had a higher surface than lower canopy leaves, but such difference was not noticeable for sessile oak leaves. Regarding the remaining parameters (**Table 1**), similar trends were observed for both species, with lower canopy leaves having a higher SLA (more marked in the case of beech), and lower leaf thickness and stomatal densities compared to top canopy leaves.

**Table 1:** Specific leaf area (SLA), area of a single leaf, thickness and stomatal density of beech and sessile oak leaves collected from upper (top) or lower (bottom) tree canopy positions.

Species	Canopy position	SLA (m^2^kg^-1^)	Area of 1 leaf (cm^2^)	Leaf thickness (μm)	Stomatal Density (N° stoma mm^-2^)
Beech	Top	14.3 ± 1.7b	72.0 ± 24.0a	219 ± 47a	216 ± 36a
	Bottom	42.2 ± 3.5a	37.0 ± 6.0b	142 ± 11b	114 ± 23b
Oak	Top	7.3 ± 1.7b	75.0 ± 11.0a	254 ± 22a	264 ± 86a
	Bottom	14.9 ± 0.7a	74.4 ± 9.6a	166 ± 8b	146 ± 83b

*Data are means ± SD (n = 30). Within columns and for the same species, values marked with different letters are significantly different according to Duncan’s Multiple Range Test (P ≤ 0.05).*

### Leaf Structure and Topography

**FIGURE 2:**
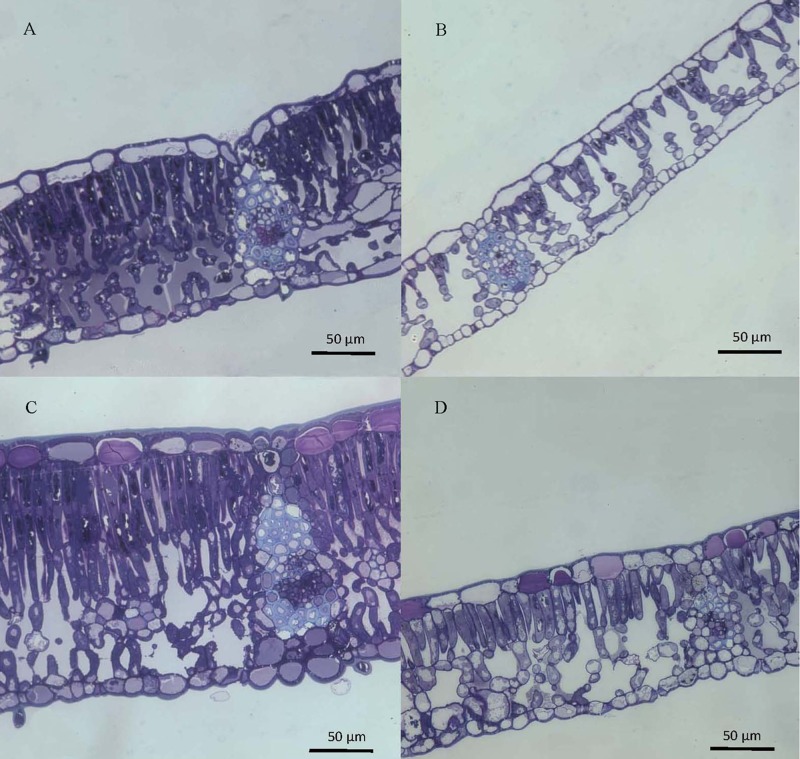
Cross-sections of beech **(A,B)** and sessile oak **(C,D)** leaves, collected from upper **(A,C)** or lower **(B,D)** tree canopy positions, stained with toluidine blue and observed by optical microscopy.

**FIGURE 3:**
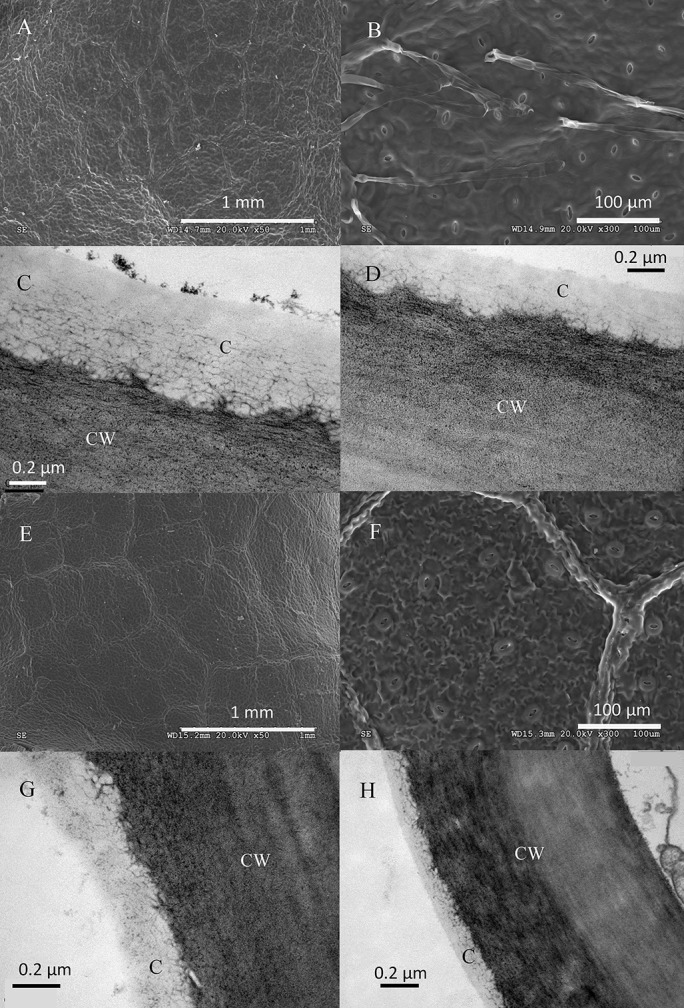
Electron micrographs of adaxial and abaxial beech leaf surfaces. SEM images correspond to: **(A)** adaxial surface of an upper canopy leaf, **(B)** abaxial upper canopy leaf, **(E)** adaxial surface of a lower canopy leaf, **(F)** abaxial surface of a lower canopy leaf. TEM micrographs correspond to: **(C)** adaxial epidermis of an upper canopy leaf, **(D)** abaxial epidermis of an upper canopy leaf, **(G)** adaxial epidermis on a lower canopy leaf, **(H)** abaxial epidermis of a lower canopy leaf.

**FIGURE 4:**
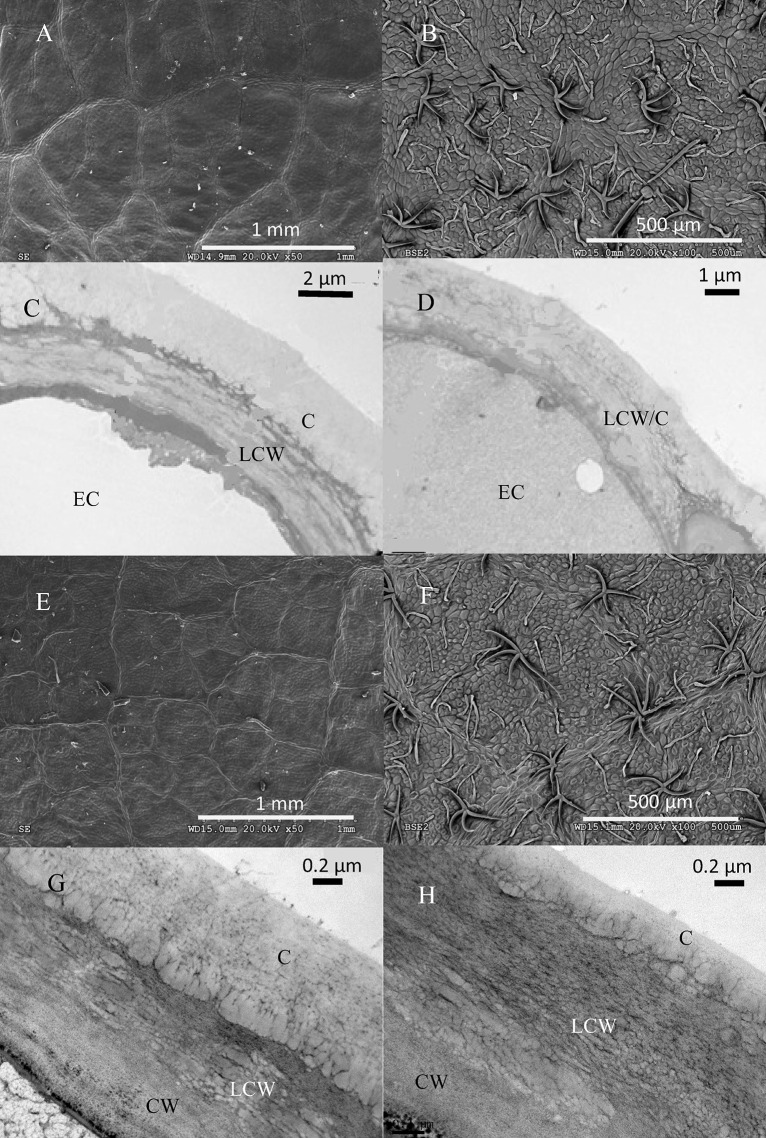
Electron micrographs of adaxial and abaxial sessile oak leaf surfaces. SEM images correspond to: **(A)** adaxial surface of an upper canopy leaf, **(B)** abaxial upper canopy leaf, **(E)** adaxial surface of a lower canopy leaf, **(F)** abaxial surface of a lower canopy leaf. TEM micrographs correspond to: **(C)** adaxial epidermis of an upper canopy leaf, **(D)** abaxial epidermis of an upper canopy leaf, **(G)** adaxial epidermis on a lower canopy leaf, **(H)** abaxial epidermis of a lower canopy leaf.

The structure of top and bottom canopy leaves of beech and sessile oak trees as observed in cross sections (**Figure 2**), is different in terms of epidermal and parenchyma cell numbers and sizes, and also regarding the air space area which was more apparent in the case of beech (**Figure 2B**). When looking at the structure of the veins, it is remarkable that top canopy beech leaves have a concave topography on the adaxial leaf side (**Figure 2A**) which is not so evident on the smaller-sized, lower canopy leaves. The topography and epidermal cell wall cross-section of the adaxial and abaxial surface of upper versus lower canopy leaves is shown in **Figure 3** for beech and **Figure 4**, for sessile oak leaves. The main difference observed concerning the surface features of beech and sessile oak leaves are the lower stomatal densities of bottom canopy leaves. Trichomes are present on the lower leaf side of both species, but no significant differences were determined in relation to different tree canopy heights (data not shown). However, a major reduction in the thickness of the adaxial and abaxial cell wall and the cuticle (interpreted as a continuous electron-lucent, whitish layer covering the external leaf area) was observed in lower canopy beech and sessile oak leaves (**Figures 3**, **4** and **Table 2**). In spite of the mentioned thickness reductions, the adaxial and abaxial cuticle of beech leaf is visible a distinct, continuous electron lucent layer (**Figure 3**). The epidermal cell wall of the sessile oak leaf is more unusual, because in addition to a continuous external electron lucent layer on the outer surface chiefly visible on the adaxial side (**Figure 4C**), it counts on an apparently lipidised cell wall (containing electron-lucent material which may be related to lipids such as, e.g., waxes) having a light-gray to whitish color, in contrast to the common electron-dense, darker appearance of polysaccharide cell walls (as observed in e.g., **Figure 3**, for beech leaves). The abaxial epidermis of top canopy sessile oak leaves have an electron-lucent material which expands to the vicinity of the epidermal cell (**Figure 4D**). The thickness of this abaxial surface is drastically reduced in leaves grown at lower canopy positions (**Figure 4H** and **Table 2**). Considering the concentration of soluble cuticular lipids (waxes), higher concentration can be observed for sessile oak compared to beech, and for the upper leaf side of both species versus the lower leaf side. For both species and leaf sides, higher soluble lipid concentrations were recovered for leaves collected from the top canopy of trees (**Table 2**).

**Table 2:** Adaxial and abaxial epidermal cell wall and cuticle thickness, and soluble cuticular lipids concentration per unit area of beech and sessile oak leaves collected from upper (top) or lower (bottom) canopy positions.

Species	Leaf side	Canopy position	Cell wall thickness (μm)	Cuticle thickness^∗^ (μm)	Soluble cuticular lipids (μg cm^-2^)
Beech					
	Adaxial	Top	3.9 ± 0.27a	0.82 ± 0.12a	32.5 ± 3.7a
		Bottom	1.8 ± 0.12b	0.26 ± 0.04b	19.4 ± 2.1b
	Abaxial	Top	2.3 ± 0.10a	0.34 ± 0.06a	22.7 ± 1.6a
		Bottom	1.2 ± 0.04b	0.10 ± 0.08b	11.3 ± 2.4b
Oak					
	Adaxial	Top	6.0 ± 0.89a	2.80 ± 0.21^∗^a	69.9 ± 4.1a
		Bottom	2.7 ± 0.30b	1.12 ± 0.17^∗^b	58.9 ± 3.2b
	Abaxial	Top	3.4 ± 0.31a	2.78 ± 0.29^∗^a	64.6 ± 1.3a
		Bottom	1.7 ± 0.37b	0.49 ± 0.32^∗^b	42.6 ± 3.1b

*Data are means ± SD. Within columns and for the same species and leaf side, values marked with different letters are significantly different according to Duncan’s Multiple Range Test (P ≤ 0.05). ^∗^Considering the continuous electron-lucent layer underneath the epicuticular waxes.*

### Contact Angles, Surface Free Energy and Related Parameters

The contact angles of the different leaf surfaces with water, glycerol and diiodomethane are shown in **Table 3**, either considering leaf blade zones excluding major veins or larger veins of the lower leaf side. The concave topography of the principal veins of the adaxial leaf side did not facilitate the accurate determination of contact angles, but such material appeared to be at least more wettable for water as compared to the surrounds leaf blade tissue (**Table 3**). For both species, the lower leaf side (abaxial) was found to be more unwettable for water, with considerably high contact angle values of water and glycerol drops measured for the abaxial side of sessile oak leaves (from 135 to 140°). For both species, the surface of the veins was found to be more wettable for the 3 liquids than the surrounding leaf blade areas. When comparing top versus lower canopy leaf surfaces, differences were chiefly found for the adaxial side of both species.

**Table 3:** Contact angles of water (θ_w_), glycerol (θ_g_) and diiodomethane (θ_d_) with adaxial and abaxial lamina and vein surfaces of beech and sessile oak leaves collected from upper or lower tree canopy positions.

Species	Leaf side	Canopy position	*θ_w_* (°)	*θ_g_* (°)	*θ_d_* (°)
Beech					
	Adaxial	Top	90.6 ± 6.4a	69.7 ± 6.5b	56.4 ± 4.2b
	Adaxial	Top, veins^∗^	70.3 ± 4.9b	–	–
	Adaxial	Bottom	95.3 ± 5.4a	88.7 ± 6.7a	68.8 ± 5.3a
	Abaxial	Top	101.8 ± 6.0a	84.9 ± 8.8a	67.4 ± 4.8a
	Abaxial	Top, veins^∗∗^	70.4 ± 5.6b	68.0 ± 5.5b	52.3 ± 3.8b
	Abaxial	Bottom	100.9 ± 5.0a	90.1 ± 5.6a	71.3 ± 5.1a
Oak					
	Adaxial	Top	104.1 ± 7.6a	83.5 ± 6.9b	66.3 ± 5.9b
	Adaxial	Bottom	87.2 ± 6.4b	95.8 ± 4.9a	72.1 ± 6.2a
	Abaxial	Top	140.3 ± 4.3a	137.2 ± 4.3a	120.8 ± 3.7a
	Abaxial	Top, veins^∗∗^	111.0 ± 4.6b	106.5 ± 5.9b	87.3 ± 4.8c
	Abaxial	Bottom	134.5 ± 6.0b	138.7 ± 5.0a	113.0 ± 5.4b

*Data are means ± SD (n = 30). Within columns and for the same species and leaf side, values marked with different letters are significantly different according to Duncan’s Multiple Range Test (P ≤ 0.05). ^∗^Veins having a slightly concave topography that invaginates into the lamina surface. ^∗∗^Veins having a prominent convex topography compared to the surrounding lamina tissue.*

When calculating the surface free energy and its components, it can be observed that the adaxial and abaxial green leaf blade areas of beech are within a range of 21 to 31 mJ m^-2^ (**Table 4**), such surfaces being mostly smooth and covered with waxes with no clear structure. The upper leaf side of sessile oak leaves collected from the top of the canopy has surface free energy values similar to that of beech leaves, which are higher for leaves collected from base of the trees. The total surface free energy of the lower side of sessile oak leaves is markedly lower chiefly due to the low wettability of such surface by polar water and glycerol. Compared to the surrounding green areas of the underside of beech and sessile oak leaves, slightly higher total surface free energy and solubility parameter values were determined for the surface of veins (**Table 4**). Regarding the dispersive component (also known as apolar or Lifshitz-van der Waals component), the highest values were recorded for the upper leaf side of top canopy leaves of both species followed by the abaxial side of beech leaf, having all the surfaces a rather smooth topography. This value was estimated to be low for the extremely unwettable sessile oak abaxial leaf side regardless of the canopy position. On the other hand, a higher

**Table 4:** Surface free energy per unit area.

Species	Leaf side	Canopy position	*γ^LW^* (mJ m^-2^)	*γ ^-^* (mJ m^-2^)	*γ^+^* (mJ m^-2^)	*γ^AB^* (mJ m^-2^)	*γ* (mJ m^-2^)	*δ (MJ^1/2^ m^-3/2^)*
Beech								
	Adaxial	Top	30.47	0.03	3.45	0.64	31.11	16.35
	Adaxial	Bottom	20.98	6.60	0.07	1.32	22.30	12.73
	Abaxial	Top	24.15	0.02	1.05	0.32	24.48	13.66
	Abaxial	Top, veins	27.27	24.48	0.02	1.50	28.77	15.41
	Abaxial	Bottom	20.81	1.93	0.06	0.66	21.47	12.38
Oak								
	Adaxial	Top	25.70	0.47	2.09	1.98	27.68	14.98
	Adaxial	Bottom	16.21	36.21	4.77	26.28	42.48	20.65
	Abaxial	Top	2.77	0.48	0.32	0.78	3.55	3.71
	Abaxial	Top, veins	12.41	3.86	0.35	2.34	14.75	9.34
	Abaxial	Bottom	3.85	3.97	2.73	6.58	10.43	7.20

*Lifshitz van der Waals component (γ^LW^), Acid-base component (γ^AB^) with the contribution of electron donor (γ^-^) and electron acceptor (γ^+^) interactions, total surface free energy (γ) and polarity (γ^AB^ γ^-1^) of beech and sessile oak leaves collected from upper (top) or lower (bottom) tree canopy positions.*

acid-base component (also known as non-dispersive component that includes polar interactions) was generally estimated for lower canopy leaves which was strikingly higher for the abaxial leaf side of sessile oak.

The solubility parameter value of all the surfaces having a rather smooth topography (i.e., both sides of beech and the upper side of sessile oak; **Figures 3**, **4**) was between 12 and 20 MJ^1/2^ m^-3/2^, with lower values being calculated for the almost super-hydrophobic, abaxial leaf surface of sessile oak.

### Foliar Absorption of Calcium Chloride

The tissue Ca concentrations of leaves treated with 3–4 μl drops of 150 mM CaCl_2_ through the adaxial or abaxial surface, are shown in **Table 5**, also in relation to untreated leaves. Due to their concave topography and larger size, only the major veins of the adaxial leaf side of top canopy leaves were treated with 4 μl drops (note the larger volume used compared to contact angle measurements, otherwise it was unfeasible to deposit the drops onto some areas of the abaxial sessile oak leaf surface due to repellency), since they enabled the deposition of the CaCl_2_ drops without falling off onto the annex green leaf blade areas. At the end of the experimental period (i.e., 24 h after drop deposition), high contact angle drops of a smaller size still remained onto the abaxial surfaces of sessile oak, while they were totally dry on the remaining treated beech and adaxial sessile oak surfaces. For top canopy beech leaves, increased leaf tissue Ca concentrations were determined after the deposition of CaCl_2_ drops onto the veins followed by the green blade areas of the upper leaf side, compared to untreated and abaxial-side treated leaves. No significant tissue Ca differences were recorded for foliar CaCl_2_-treated sessile oak leaves and lower canopy beech leaves (**Table 5**).

**Table 5:** Leaf tissue Ca concentrations (g 100 g^-1^ dry weight, D.W.) 1 day after application of 2 drops cm^-2^ of 150 mM CaCl_2_ onto the lamina of adaxial or abaxial side of beech and sessile oak leaves, collected from upper (top) or lower (bottom) tree canopy positions.

	Tissue Ca concentration (g 100 g^-1^ D.W.)			
Treatment	Beech		Oak	
	Top	Bottom	Top	Bottom
Untreated	0.59 ± 0.06b	0.86 ± 0.01a	0.82 ± 0.04a	0.82 ± 0.06a
Adaxial	0.67 ± 0.06ab	0.89 ± 0.02a	0.86 ± 0.08a	0.80 ± 0.07a
Abaxial	0.58 ± 0.05b	0.87 ± 0.02a	0.79 ± 0.04a	0.85 ± 0.13a
Veins^§^	0.71 ± 0.05a			

*Data are means ± SD (n = 3). Within columns and for the same species and canopy positions, values marked with different letters are significantly different according to Duncan’s Multiple Range Test (P ≤ 0.05). ^§^Drops were deposited onto the veins adaxial leaf side.*

Concerning the Ca and Cl concentrations recovered in the wash of CaCl_2_ treated (data not shown) and untreated sessile oak and beech leaves (**Table 6**), strikingly high Cl concentrations were cleared from the surface of untreated leaves collected from the top of the trees compared to those located at lower canopy positions.

**Table 6:** Calcium and Cl concentration (μM cm^-2^) washed from the surface of untreated beech and sessile oak leaves collected from upper (top) or lower (bottom) tree canopy positions.

Species	Canopy position	[Ca] (μM cm^-2^)	[Cl] (μM cm^-2^)
Beech	Top	0.19 ± 0.09a	58.44 ± 1.36a
	Bottom	0.10 ± 0.06a	3.27 ± 0.22b
Sessile oak	Top	0.13 ± 0.10a	59.99 ± 1.19a
	Bottom	0.09 ± 0.08a	4.09 ± 0.12b

*Data are means ± SD (n = 3). Within columns and for the same species values marked with different letters are significantly different according to Duncan’s Multiple Range Test (P ≤ 0.05).*

## Discussion

In this study, a comparison between leaves growing on the same tree but at different heights was established with focus on plant surface properties. For both species, leaf burst is first observed to occur in top canopy shoots, with leaves developing in lower canopy positions a few days later (e.g., from 3 to 7 days depending on the prevailing weather conditions; G. González Gordaliza, personal communication). It is hence assumed that the leaves sampled in this study were of a similar age, and that lower canopy leaves always developed under average lower irradiation conditions, including also the effect of diurnal variations and weather conditions during the growing season.

A major reduction on cuticle and the epidermal cell wall thickness and wax load per unit surface, was recorded for lower canopy leaves collected by mid July 2016. When considering the major environmental factors influencing such structural variations on beech and sessile oak leaves of the same individuals, comparatively less marked temperature and relative humidity variations were recorded between the top and the lower canopy of trees, compared to irradiation which is potentially the main factor triggering the epidermal modifications observed as noted by other authors (e.g., Aranda et al., 2001; Cano et al., 2013). Increased irradiation has been found to raise epicuticular wax amounts in several species (e.g., Tevini and Steinmüller, 1987; Shepherd and Wynne Griffiths, 2006). However, this is the first study in which an effect of variable light exposure (among other climatic factors) in the same individual and organ kind is described to cause such major effects on epidermal cell wall and cuticle thickness. The prevailing environmental conditions in the lower part of the tree canopy drastically affected the development of the leaf cuticle of sessile oak (40 and17.6% reduction for the adaxial and abaxial leaf surface) and beech (32 and 29.4% reduction for the adaxial and abaxial leaf surface, respectively). The epidermal cell wall thickness (including the cuticle) of lower canopy leaves decreased by 46 and 52% for the adaxial and abaxial leaf side of beech, and by 45 and 50% for the adaxial and abaxial leaf side of sessile oak. The unusual nature of the sessile oak leaf cuticle compared to cuticles from other species assessed so far (see Jeffree, 2006) should be further examined with regard to its structure and composition.

When analyzing leaf structural changes of beech leaves of different irradiation levels collected from trees also grown at ‘Montejo de la Sierra Forest,’ Aranda et al. (2001) and Cano et al. (2013) noted the great capacity of beech to acclimate to low irradiation conditions that involves anatomical changes such as an increased the SLA, reduced blade thickness, and lower stomatal densities as also observed in our study for lower canopy leaves. Similar anatomical modifications in response to light were also reported by Barna (2004) for beech leaves collected at different canopy heights in Slovakia. Working at the same location under a similar experimental setting, Cano et al. (2013) evaluated the response of beech and sessile oak leaves from different canopy levels to summer drought, chiefly focusing on leaf photosynthesis. In response to moderate summer drought, they observed significant photosynthesis rate decreases in top and low canopy beech leaves in July. Concerning sessile oak trees, the maximum gas exchange rate of top canopy leaves was reached in August (i.e., after a longer period of summer drought compared to July), while low canopy leaves experienced a continued gas exchange decrease from July on (i.e., after a moderate drought period). The authors reported that the gas exchange reductions observed in response to drought were related to a decrease in stomatal and mesophyll conductance, with stomatal limitations being more significant in leaves from the top canopy, and mesophyll limitations being larger in leaves from the lower crown area (Cano et al., 2013).

In this study, we focused on the leaf surface properties of top versus low canopy leaves of both species, to assess traits that may minimize water loss at the higher temperatures and vapor pressure deficits occurring at the upper canopy which may constrain carbon fixation even in the absence of water stress (Niinemets, 2007). We observed increased amounts of soluble cuticular waxes and thicker epidermal cell walls and cuticles in top canopy leaves compared to leaves growing at the base of the crown. Current gas exchange and hydraulic models are attempting to consider leaf anatomy traits (Terashima et al., 2001; Milla-Moreno et al., 2016; Ouyang et al., 2017), and cell wall thickness has been proposed as main factor affecting mesophyll CO_2_ conductance (e.g., Scafaro et al., 2011; Tomás et al., 2013; Peguero-Pina et al., 2017; Scoffoni et al., 2017; Veromann-Jürgenson et al., 2017). However, the significance of cuticle and epidermal cell wall thickness regarding the diffusion of gasses is currently unclear, and must be explored in future investigations. It must be highlighted that at least for the chemicals supplied in liquid form, cuticle thickness has not been found to be related to cuticular permeability (e.g., Norris, 1974; Becker et al., 1986; Knoche et al., 2000; Buchholz, 2006). On the other hand, cuticular waxes have been identified as the main barrier to transpiration, and also to the penetration of molecules such as water, ions, or organic chemicals (e.g., Riederer and Schreiber, 2001; Buchholz, 2006; Schreiber and Schönherr, 2009). The importance of intra-cuticular versus epi-cuticular waxes in limiting water loss has been examined in some studies, and it has been suggested that this may be variable depending on the species (Jetter and Riederer, 2016; Zeisler and Schreiber, 2016). However, apart from the chemical composition, the degree of crystallinity, packing and orientation of cuticular waxes as shown by Hama et al. (2017) for *Kalanchoe pinnata* leaves, may affect cuticular barrier properties and this should be considered in future studies. The unusual structure and potential composition of the sessile oak leaf cuticle and epidermal cell wall as observed in our study chiefly in relation to top canopy positions, may be related to preventing water loss but these aspects shall be analyzed in future investigations.

The significance of foliar water uptake for improving water economy due to the deposition of e.g., water onto the surfaces as rain, fog or dew is gradually receiving more scientific attention (as summarized by Fernández et al., 2017). The potential process of foliar absorption will be first influenced by plant surface-liquid interactions between water and leaf surfaces, and the subsequent transport through the plant surface (Fernández and Brown, 2013; Fernández et al., 2017). The importance of plant surface wettability has long been recognized as key factor for foliar absorption (e.g., Holloway, 1969; Smith and McClean, 1989; Brewer et al., 1991). The adaxial and abaxial side of fully expanded, mature, top and low canopy beech leaves examined in this study were found to be non-wettable for water (from 90 to 102°), which is agreement with the results of Klamerus-Iwan and Kraj (2017) for beech trees grown in Southern Poland. Both leaf sides of beech and also the adaxial side of sessile oak, are rather smooth and in general had total surface free energy (γ_s_) and solubility parameter (*δ*) values similar to those reported for rather flat and waxy leaves such as orange leaf (Fernández et al., 2017). The *δ* values estimated are not far from theoretical value calculated for model epicuticular wax compounds (i.e., 16 to 17 MJ^1/2^ m^-3/2^ for waxes; Khayet and Fernández, 2012). It is remarkable that top canopy leaves had increased wax concentrations per unit area and a higher dispersive (apolar, γ^LW)^ free energy component. In comparison with the surrounding leaf blade of beech and sessile oak, the surface of veins was more wettable for the 3 liquids. Hence, our results clearly demonstrate that the material covering the veins may be structurally and chemically different, compared to the cuticle covering green lamina areas, but this aspect must be examined more in detail in future trials.

Both leaf sessile oak surfaces were found to be more unwettable for top canopy leaves, the lower side being almost super-hydrophobic and with drop repellence in some parts as described for *Quercus ilex* (Fernández et al., 2014b). As a result of its unwettable character for the 3 liquids tested, which is likely due to its roughness, the abaxial leaf surface of sessile oak has a low total γ_s_ and *δ* which is below the theoretical value of epicuticular waxes (i.e., 16 to 17 MJ^1/2^ m-^3/2^ for waxes; Khayet and Fernández, 2012). Such low values have also been recorded for highly unwettable surfaces, such as blue gum eucalypt, wheat or holm oak leaf (Fernández et al., 2014a,b, 2017; Fernández and Khayet, 2015).

From the contact angles of the 3 liquids measured, we could a prior hypothesize that foliar absorption may occur through both beech leaf surfaces including the veins, and may be also via the upper leaf side of sessile oak, foliar penetration via the abaxial surface of latter species being unlikely to occur due to the low contact angles, drop repellence and low γ_s_ determined experimentally.

The application of foliar sprays in agriculture has been applied since the XIX Century and many efforts were carried out during the last decades chiefly to characterize the permeability of isolated, stomatous leaf cuticles from few species (Fernández and Eichert, 2009). The process of foliar absorption may take place via the cuticle, stomata, irregularities in the cuticle, trichomes or other epidermal structures (Fernández et al., 2017). In light of cuticular permeability trials, it has been hypothesized that lipophilic molecules should diffuse through the more lipophilic domain of the hydrophobic cuticle (Niederl et al., 1998; Buchholz, 2006), while small, polar molecules such as water may follow a different diffusion pathway which is currently not well characterized and has been associated with a “dynamic aqueous continuum” forming due to hydration (Fernández et al., 2017), and to “polar” or “aqueous” pores (Franke, 1967; Schönherr, 2006; Tredenick et al., 2017) in the cuticle, whose occurrence has never been directly demonstrated so far (Fernández et al., 2016, 2017).

Calcium chloride has been used in several cuticular permeability trials as active ingredient for characterizing the transport of electrolytes (e.g., Schönherr, 2000, 2001; Schlegel and Schönherr, 2002). We hence, used this salt as tracer for the potential absorption of water, applying a new approach for the systematic comparison of CaCl_2_ transport through sessile oak and beech leaf surfaces. For this purpose, 2 drops per cm^2^ of this liquid were deposited onto the adaxial and abaxial side of beech and sessile oak leaves collected from the 2 different canopy locations. Due to their topography and larger size, drops were also deposited onto the major veins of the adaxial side of top canopy beech leaves, and this was actually the treatment that led to increased tissue Ca concentrations. Higher tissue Ca concentrations were also recorded after treating the adaxial side of top canopy beech leaves, the remaining results being statistically similar to untreated beech and sessile oak leaves. While applying 2 drops per cm^2^ in all leaf surfaces enabled us to establish exact comparisons between treatments, the system proved extremely time-consuming and made us reduce the number of treated leaves. Hence, similar trials under different relative humidity and temperature conditions should be carried out in the future for estimating the absorption of solutes by different leaf lamina areas. Thereby, it is concluded that at least the top canopy beech leaves may absorb water via the upper leaf side but future trials should be carried out for assessing the potential contribution of foliar water absorption to water economy chiefly under drought conditions, as reported by Pflug et al. (2018).

Last but not least, when analyzing the wash after the foliar application experiment, large amounts of Cl that were removed from the surface of top canopy sessile oak and beech leaves and not from leaves from the bottom of the crown. It is commonly accepted that the primary source of the atmospheric chloride comes from the sea, but anthropogenic sources may add Cl to the atmosphere via dry deposition or wet deposition (Guan et al., 2010). Several reports showed that chloride deposition was much higher in a forest canopy than in the adjacent open fields regardless of vegetation species and climate types (Deng et al., 2013). This phenomenon has been often related to dry deposition intercepted by forest canopies (Beier et al., 1993).

On the other hand, it appears unlikely that Cl was extruded from the interior to the external surface of leaves located at the top of the canopy, but the reason behind the high Cl concentration recovered from the surface of top crown leaves in ‘Montejo de la Sierra Forest’ should be investigated more in detail in the future.

## Conclusion

In this study, we analyzed the surface properties of sessile oak and beech leaves collected either from the top or the bottom of the crown of trees growing at ‘Montejo de la Sierra Forest.’ In general, leaves from the top canopy had increased cuticular wax concentrations per unit area, and were less wettable. The lower leaf side of sessile oak was almost super-hydrophobic, and it was found *a priori* unlikely to be permeable to water and solutes. Leaf surfaces were observed to have at least 2 distinct lamina zones: the surface of veins and the green areas covered with a more homogeneous cuticle. While vein topography varied according to the upper or lower leaf side, the surface of veins appeared to be more wettable and enabled the absorption of CaCl_2_ by top canopy beech leaves. It is concluded that the mechanisms of foliar absorption must be further characterized by examining e.g., leaves from different species, cultivars and growing conditions. Emphasis should be made on the role of surface features of the adaxial and abaxial surface such as veins, trichomes or stomata, in addition to cuticular composition and structure as pre-requisite for suggesting broad foliar permeability models.

## Author Contributions

HB contributed to designing the study, developing the experiments, analyzing and interpreting the results, writing the draft, and approving its final version. VF participated in the study design, development and interpretation of the results, also contributing to writing, revising, and approving the final draft. LG contributed to discussions for the development of the experiments and the interpretation of the results. He also revised the manuscript and approved its final version.

## Conflict of Interest Statement

The authors declare that the research was conducted in the absence of any commercial or financial relationships that could be construed as a potential conflict of interest.
